# Supplementary material for: Exploring the potential for children to act on antimicrobial resistance in Nepal: Valuable insights from secondary analysis of qualitative data
Source: PLoS One. 2023 Jun 2;18(6):e0285882. doi: 10.1371/journal.pone.0285882 (PMC10237405; doi:10.1371/journal.pone.0285882)
Supplement: S1 File — (DOCX) [file pone.0285882.s001.docx]

***Exploring the potential for children to act on AMR in Nepal: valuable insights from secondary analysis of qualitative data***

**S1 File - Transcript inventory – selected transcripts used in the paper**

| **Transcript name and number (shortcut used in the paper)** | **Search term** | **Transcript** | **Context of the transcript data (where applicable)** |
| --- | --- | --- | --- |
| Workshop 3 transcript: Chandragiri  (W3) | Child*  School  Student | **Moderator (M):** There is the practice of using the same medicines for the livestock amongst the general people. They say that my cow got better using this medicine so you can take this.  **Female participant (F):** They will hand each other the remaining medicines hoping that it will help the other person’s cow to get better as well.  **M:** And do they do the same when it comes to **children** as well?  **All the participants:** No, they do not do so when it comes to **children**.  **M:** Why do you think that they do this for the adults but not for the **children**?  **F:** Maybe it is because they love their **children** more.  **F:** The **children** are weak.  **F:** They are small and young.  **M:** Yes.  **F:** They think that the **children** are very young and that it might harm them. It might not work on **children** but it will be useful amongst the adults. They think that the **children** might have to suffer in case the dose is not correct for them.  **I1:** You think that the **children** are weaker than the adults while the adults can get better using the same medicines that other adults are using? That is the mentality that the people here have?  **F:** Yes. | N/A |
| Focus Group Discussion 5 with workshop participants post film showcasing  (FGD 5) | Child* | **M:** Does that mean you used to self-administer antibiotics without any medical consultation? Can you tell me about your prior practice of antibiotics use?  **P7:** I used to self-determine the antibiotics for the illness that I experienced. And I used to buy them and take them on my own. I even used to instruct my **children** to buy the medicines for me. | Child purchasing antibiotics |
| Semi Structured interview 5 Chandragiri with local stakeholder (SSI 5) | Child*  School | **P:** [Interrupting] If we can specifically take it to the **school** sectors then these messages will travel to the community faster. Each household will get this information from there. … I think that almost all the households within this municipality will get the message that it gives. That is because every guardian has a **child** that goes to one of these schools. Plus, there are teachers there as well.  **M:** Yes.  **P:** The teachers will also explain these things to their students so that they can understand it better. Each household will then get that message. Along with that, we could also provide them with a small pamphlet each so that they can take home. The people back home will also understand it. Even if we cannot distribute such materials, they will have the experience of watching the documentary and understanding the message that it gives. I think that is a faster way to raise awareness [on ABR].  **M:** All right. | In response to sharing programme more widely within the community |
| Focus Group Discussion 4 with film screening participants (FGD 4) |  | **M**: All right. …, who are the people that should be shown those films? Which sector should we also focus on?  **P2**: …We should actually focus the **children** and housewives with this program. It would be better if we could focus on them. | Wider audience for films |
| Focus Group Discussion 4 with film screening participants (FGD 4) | Child*  School  Parent  Student | **M**: And students from which grade should be shown these films?  ..  **P4:** Yes, I think that **students** from grade 1 and higher should be shown the film. Most of the families have the same practice of using the medicines again when they fall ill later. They will get some information looking at it and will go home and tell their families about it. They could tell their friends and family back in the village about it which would be good. | Discussions on age of children to be shown the film |
| Focus Group Discussion 4 with film screening participants (4 FGD) | Child*  School  Parent  Student | **M:** And students from which grade should be shown these films?  **P3**: What should I say…? It would be better if everyone was shown this film. However, the young **children** might not be able to understand it. So, I think that it would be better if we could show it to **students** that are studying in grade 1 and higher. The **children** studying in the kindergarten might not understand anything. That is all. (*Note: says shying away). | Discussions on age of children to be shown the film |
| Focus Group Discussion 4 with film screening participants (4 FGD) | Child*  Family  School  Student | **M**: So you said that your **family** members send you to get their medicines. At what age do they feel that you are able to understand what medicines they are referring to? I think that it will be easier to determine the age in this manner.  **P3**: Umm…  **P4**: When we are 10 years old.  **M**: So it would be better to show it to the **children** of ages 10 and above?  **P3:** Yes. They will be able to understand it. | Discussion feeding on from the point above but participants directly referring to being instructed to buy medicines when they were children– using this information to justify the timing of school-based Antibiotic education. |
